# Deep learning pose detection model for sow locomotion

**DOI:** 10.1038/s41598-024-62151-7

**Published:** 2024-07-16

**Authors:** Tauana Maria Carlos Guimarães de Paula, Rafael Vieira de Sousa, Marisol Parada Sarmiento, Ton Kramer, Edson José de Souza Sardinha, Leandro Sabei, Júlia Silvestrini Machado, Mirela Vilioti, Adroaldo José Zanella

**Affiliations:** 1 Department of Preventive Veterinary Medicine and Animal Health, School of Veterinary Medicine and Animal Science, Center for Comparative Studies in Sustainability, Health and Welfare, University of São Paulo, Pirassununga, SP 13635-900 Brazil; 2 Robotics and Automation Group for Biosystems Engineering, Department of Biosystems Engineering, Faculty of Animal Science and Food Engineering (FZEA), University of São Paulo (USP), Pirassununga, SP 13635-900 Brazil; 3 Zinpro Corporation, Piracicaba, SP Brazil

**Keywords:** Computational biology and bioinformatics, Image processing

## Abstract

Lameness affects animal mobility, causing pain and discomfort. Lameness in early stages often goes undetected due to a lack of observation, precision, and reliability. Automated and non-invasive systems offer precision and detection ease and may improve animal welfare. This study was conducted to create a repository of images and videos of sows with different locomotion scores. Our goal is to develop a computer vision model for automatically identifying specific points on the sow's body. The automatic identification and ability to track specific body areas, will allow us to conduct kinematic studies with the aim of facilitating the detection of lameness using deep learning. The video database was collected on a pig farm with a scenario built to allow filming of sows in locomotion with different lameness scores. Two stereo cameras were used to record 2D videos images. Thirteen locomotion experts assessed the videos using the Locomotion Score System developed by Zinpro Corporation. From this annotated repository, computational models were trained and tested using the open-source deep learning-based animal pose tracking framework SLEAP (Social LEAP Estimates Animal Poses). The top-performing models were constructed using the LEAP architecture to accurately track 6 (lateral view) and 10 (dorsal view) skeleton keypoints. The architecture achieved average precisions values of 0.90 and 0.72, average distances of 6.83 and 11.37 in pixel, and similarities of 0.94 and 0.86 for the lateral and dorsal views, respectively. These computational models are proposed as a Precision Livestock Farming tool and method for identifying and estimating postures in pigs automatically and objectively. The 2D video image repository with different pig locomotion scores can be used as a tool for teaching and research. Based on our skeleton keypoint classification results, an automatic system could be developed. This could contribute to the objective assessment of locomotion scores in sows, improving their welfare.

## Introduction

Lameness in animals affects locomotion, causing pain and discomfort^1,2^. In the Welfare Quality Assessment Protocol for pigs, project sponsored by the European Union^3^, lameness is one of the most important parameters to assess good health, as a welfare criterion to check for absence of injuries. Due to pain caused by lameness, animals reduce their activities, such as eating, drinking, walking, and socializing^4^; however, they perform more passive activities, such as sleeping and lying down^4,5^, which compromises their species-specific behaviour.

Lameness is exhibit throughout the breeding cycle of the pig and can be diagnosed in the first three weeks of the piglet’s life^6^. Lesions in the locomotor system can be related to lameness^7^. The prevalence of lameness in sows is rarely reported and is usually underestimated, with reports ranging from 8 to 65%^8,10^. In a study by Jorgensen^7^, lesions were identified in the locomotor systems of 71–99% of the sows, including surface lesions on the claw and heel, as well as inappropriate claw sizes and excessive wear on the sidewall of the claw. In another study, it was found that 96% of sows had cracks in the heel^9^. A Brazilian study showed that 99.1% of sows present claw lesions, and 89.9% of sows present heel overgrowth and erosion lesions^11^. The presence of lameness in pigs is multifactorial and impacted by genetics, fights among animals, vitamin and mineral imbalances in the diet^10,12^, the type of feed provided^1^, and, especially, concrete floor housing^13^. Furthermore, housing conditions influence hoof health^14,15^, since poor hygiene, wet floors, and high animal density can lead to secondary problems, such as lesion contamination and subsequent infections^14^.

Lameness is responsible for the unplanned culling of 11% of sows and 13% of gilts annually, resulting in estimated treatment costs between €37 and €138 per lame sow^16^. Additionally, it was estimated that between 20 and 30% of the sows that become lame during pregnancy die 10 days after farrowing^17^. Piglets born from lame sows exhibit changes in weight at weaning, increased aggressive behaviour, and altered quantity of vocalizations during emotional tests^18^. Likewise, heel lesions are associated with the presence of crushed piglets, and mummified foetuses are twice as likely to be borne from lame sows as from non-lame sows^9^. Lameness in sows results in economic losses, health and welfare problems, and negative impacts to the offspring of the sows^7,18^.

In the study by Heinonen et al.^1^, 10% of animals with lameness were either not identified as lame by their owners or did not receive treatment. Lameness often goes unnoticed in its early stages; as a result, the animals receive treatment at advanced ages^2^. It is of outmost importance to carry out a diagnosis of lameness in the early stages of the condition in order to prevent situations of stress and unnecessary pain for the animal^8^ and to promote quick recovery. Identifying sows with early signs of lameness is ideal for improving welfare and reducing economic impacts^2,19,21^.

Animal observation is necessary for identifying and treating lameness. In pig farming, observation and correct identification of lameness are often lacking due to untrained staff and time-consumption, resulting in low precision and diagnosis reliability^2^. A common method for assessing lameness in pigs is assigning a visual numerical rating score (NRS) using a locomotion scale system^20,22^. However, this method is subjective, as it depends on the training and experience of the observer, which compromises the reliability and precision of the methods^21^. Currently, objective methods for assessing lameness are being investigated by utilizing technology to yield dependable analyses, precise diagnoses, and minimal labour requirements^2,21^, such as Precision Livestock Farming (PLF) technologies.

PLF technology manages individual animals by utilizing continuous real-time monitoring, including sensors, cameras, and sound analysis^23^. This monitoring is accessible to the operator at any given time, thereby keeping the operator apprised of the animal's condition^23^. 2D colour camera (RGB) has been used in 33% of studies for assessing gait in pigs^24^. Emerging technologies aligned with the PLF paradigm enable management issues to be detected, provide more precise guidance for executing decision-making and management tasks^25^, and assist in monitoring animal health and behaviour^26^ through individual recognition^27^. As a result, it reduces labour requirements and provides nonsubjective data for evaluating and improving animal welfare indicators^25,26^. Some examples of proposed PLF technology being used with pigs are the Sow Stance Information System (SowSIS)^28,29^, automatic monitoring of pig locomotion using images^30^, automatic piglet tracking^31^, vocalization use for identifying sex, age, and distress in pigs^32^, and behavioural recognition in pigs and cattle^33^.

Pose models have also been used as Precision Livesock Farming -PLF tools with other animals, including farmed pigs, such as:^34^ proposed a pose estimation model for pigs in collective pens to detect diseases early on by observing behavior. The problem of limb occlusion, e.g. leg, elbow, and tail, can be solved using this model. In a previous work^35^, it was proposed to use the software created as a semi-automatic tool to find, identify and track poses of infrared marks and label the 3D images. Published research^36^, reported the use of pose software for automatic individual identification of pig, recording weight and activity levels.

This study was conducted with two key goals. First, a repository containing 2D videos images assessed by experts and featuring sows displaying distinct locomotion scores is established. Second, a methodology for constructing a computational model through automated machine vision systems to identify the lateral and dorsal poses of sows in locomotion is developed by employing deep learning techniques.

## Materials and methods

Data were collected in a commercial pig farm (Topgen) located in Jaguariaíva, Paraná, Brazil. The experiment was conducted with the approval of the Ethics Committee on Animal Use (CEUA) of the Faculty of Veterinary Medicine and Animal Science at the University of São Paulo (USP) under registration number 9870211117. The study was conducted according to the ARRIVE guidelines (https://arriveguidelines.org/). Vision computer models were created through support by the Robotics and Automation Group for Biosystems Engineering (RAEB) at the Faculty of Animal Science and Food Engineering of the USP.

### Animal experimentation and data acquisition

A sample of 500 sows in locomotion (Landrace × Large White, Afrodite line) was used to individually record 2D videos images (it has two dimensions: width and length) to create the video image repository.

The farm's routine was not altered for this experiment. Data was collected every day between 8 a.m. and 5 p.m. from May 9th through May 17th, 2022.

The filming setup was built in an empty pen (6 × 4 m) in the farm facilities. A solid floor area was delimited with two galvanized wires (2.10 mm) to create a corridor. At the end of the corridor, a return area was provided for the animals. The corridor and the wall were painted with white acrylic wall paint to enhance the contrast between the animals and the setting. The filming area measured 1.5 m wide by 5 m long.

**Figure 1:**
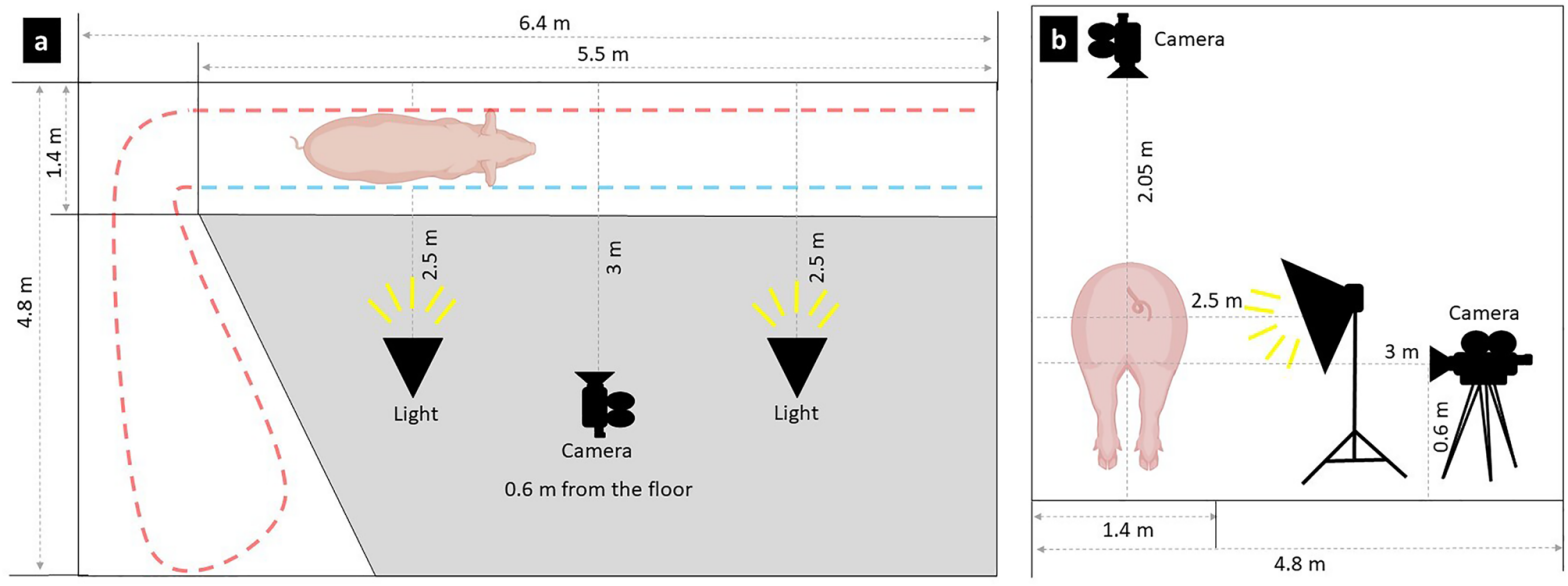
Scenario used to collect the data of sows in locomotion. (**a**) superior view, and (**b**) lateral view. The red dashed line is the animal's route of entering and turning around. The blue dashed line is the animal's route out of the pen.

**Figure 2:**
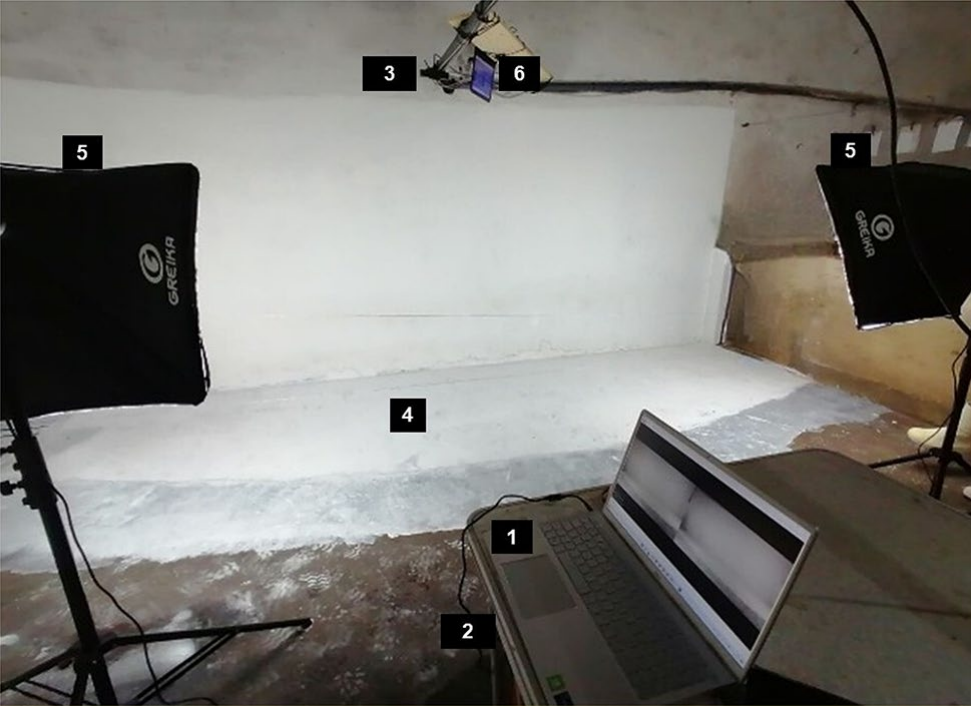
Scenario and equipment used to collect the data of sows in locomotion using lateral and dorsal views. (1) laptop (Core i7); (2) under the table Zed i2 camera and (3) along with the feed tube; (4) corridor where the animals walked; (5) Greika PK-SB01 lighting kit; and (6) laptop (Core (TM) i5).

The equipment was strategically chosen as follows: a Dell Inspiron 15 5502 laptop (featuring Core i7, Microsoft Windows 11, 16 GB RAM, 512 GB SSD, NVIDIA GeForce) was used to record lateral videos, and a Dell Inspiron 3421 laptop (featuring Core (TM) i5, Microsoft Windows 10, NVIDIA Geforce) was used to record dorsal videos. A ZED 2i Stereo Camera (Stereolabs Inc., USA) was positioned 0.6 m from the floor and 3 m away from the corridor wall where the animals passed. Another camera was positioned above the corridor at 2.05 m from the floor. This configuration yielded comprehensive lateral and dorsal views of the entire length of the animals, extending from the cranial to caudal. To regulate the luminance of the environment and enhance the video quality, an artificial light system was placed on each extreme of the corridor behind the wires and 2.5 m away from the corridor wall (Greika PK-SB01 lighting kit, two 50 × 70 cm soft boxes). Figures 1 and 2 illustrate the data acquisition facilities.

The ZED 2i camera was configured to capture RGB images and point clouds in HD (1920 × 1080 pixels) at 15 frames per second, with an average duration of 30 s per video per animal.

### Video preprocessing and annotation

The filtering process had two stages: the sow entering the corridor and the sow passing through the corridor, as illustrated in Fig. 1. Only the returning part was used for training the models (right lateral side of the animal and the entire dorsal side of the animal). A Python script was used to remove the moment when the animal leaves the pen. The video was then converted from SVO to MP4 format.

**Figure 3:**
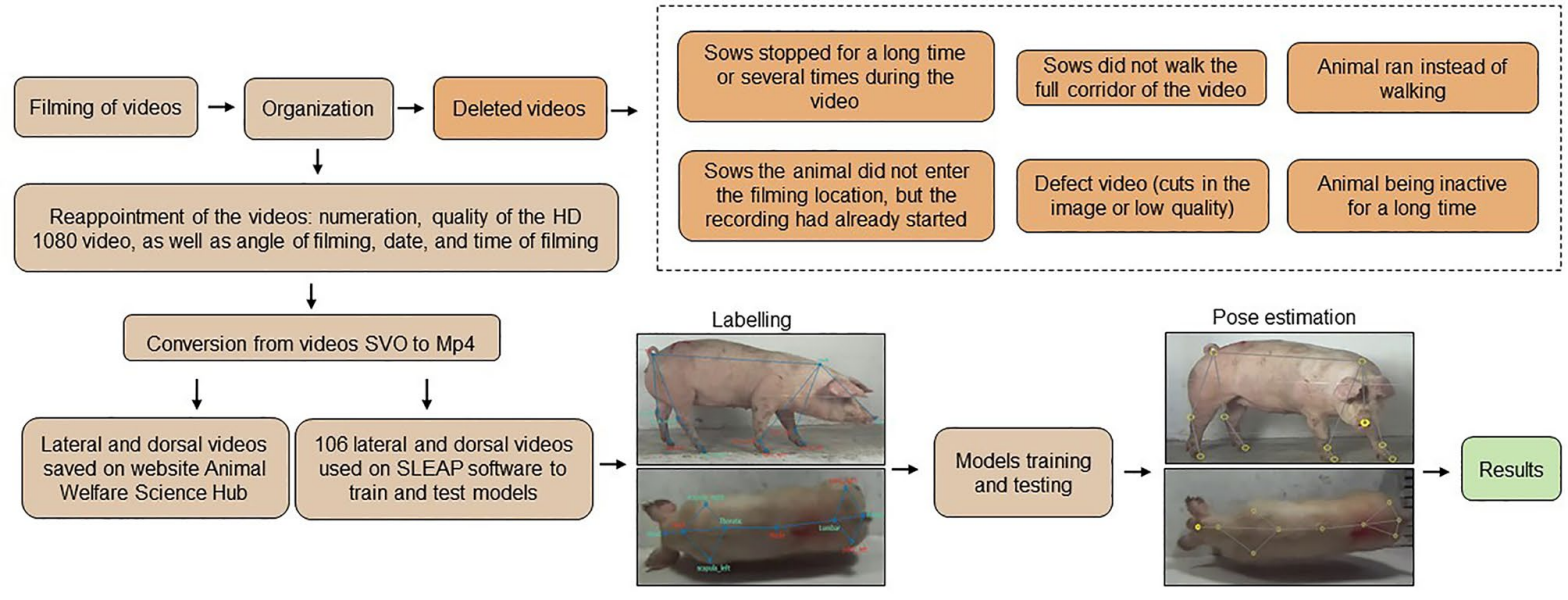
Workflow chart of the proceorganizing videos to add to SLEAP software and save on the website Animal Welfare Science Hub, as well as the training and testing models and results.

A total of 1207 videos, 565 lateral and 642 dorsal videos, were recorded; however, 40% of the videos were not used due to issues encountered during filming. Some problems included the sow not walking, being inactive for a long time, or running instead of walking, and video defects such as cuts in the image or low image quality. A total of 364 lateral and 336 dorsal videos were converted to the MP4 format (Fig. 3).

Thirteen experts in farm animal locomotion assessment categorized each sow video using the Zinpro Swine Locomotion Scoring system. The scores ranged from 0 to 3, from no signs of lameness to severe lameness (Table 1).

**Table 1:** Zinpro’s Swine locomotion scoring system to assess lameness in pigs.

Scores	Description
0	No signs of lameness. The animal moves easily with little inducement and is comfortable on all its feet
1	The animal moves relatively easily, but there are visible signs of lameness in at least one leg. The animal is reluctant to bear weight with that leg, but it still moves easily between sites in the barn
2	The animal exhibits lameness in one or more limbs and compensatory behaviours such as dipping of the head or arching of the back
3	The animal shows real reluctance to walk or to bear weight on one or more limbs. As a result, moving the animal between places on the farm is difficult

The 364 lateral videos were evaluated by the experts using Google Forms. Only the lateral view was assessed by the experts. After the 364 videos were analysed, 11 videos were removed because the experts were unable to classify the animals' locomotion scores due to the sows slipping at the beginning of the video, making it difficult for the experts to assess locomotion. Scores indicated by more than 50% of the experts were considered as the final score. In addition, descriptive statistical analysis through the mean, median, standard deviation, maximum and minimum with box plot visualization was performed to identify outlier experts, resulting in the removal of three experts and their respective responses. The statistical analysis was performed using Jamovi^37^ and R^38^ software.

In the SLEAP software (Social LEAP Estimates Animal Poses), only lateral videos that had the corresponding dorsal view were used. The score assigned to each lateral view video was also assigned to the corresponding dorsal view video for each sow. During the video evaluations for determining locomotion scores, there were divergent responses. Therefore, the differences between the scores were calculated using Microsoft^®^ Excel, after which the degree of certainty of the answer was calculated. To calculate the differences between the answers (DBA), the maximum function is added to the range of locomotion scores (0, 1, 2 and 3); the range of locomotion scores is subtracted from this; and the maximum is then added again. The formula for calculating the DBA demonstrated for the first time is as follows (1):

1 $$DBA=max\left(loc \,scores\right)-(sum\left( loc \,scores\right)-{\text{max}}(loc \,scores))$$

After calculating DBA, the response confidence, provided in Table 2, was calculated by applying if–then functions. If the DBA is less than − 1, then there is no confidence in the score evaluation. If the DBA is less than 0, then there is 25% certainty in the score evaluation. If the DBA is less than 2, then there is 75–100% certainty in the score evaluation.

**Table 2:** Degree of reliability of the evaluation by lameness experts of the sows' locomotion scores.

Calculation of difference between answers	Calculation of confidence in answering
< (− 1)	0%
< (0)	25%
< (1)	75%
< (2)	100%

### Computer vision models

The computational models were constructed and tested for different deep learning architectures. The processing of the models was carried out using an HP Z2 Tower G5 workstation computer with an Intel Xeon W-1270 CPU, 32 GB RAM, Windows 10 Pro for Workstations version 21H2, and the tool SLEAP (version 1.3.0). SLEAP is an open-source software developed in the Python programming language that provides a deep learning-based framework for pose estimation in different animal species^39^.

**Figure 4:**
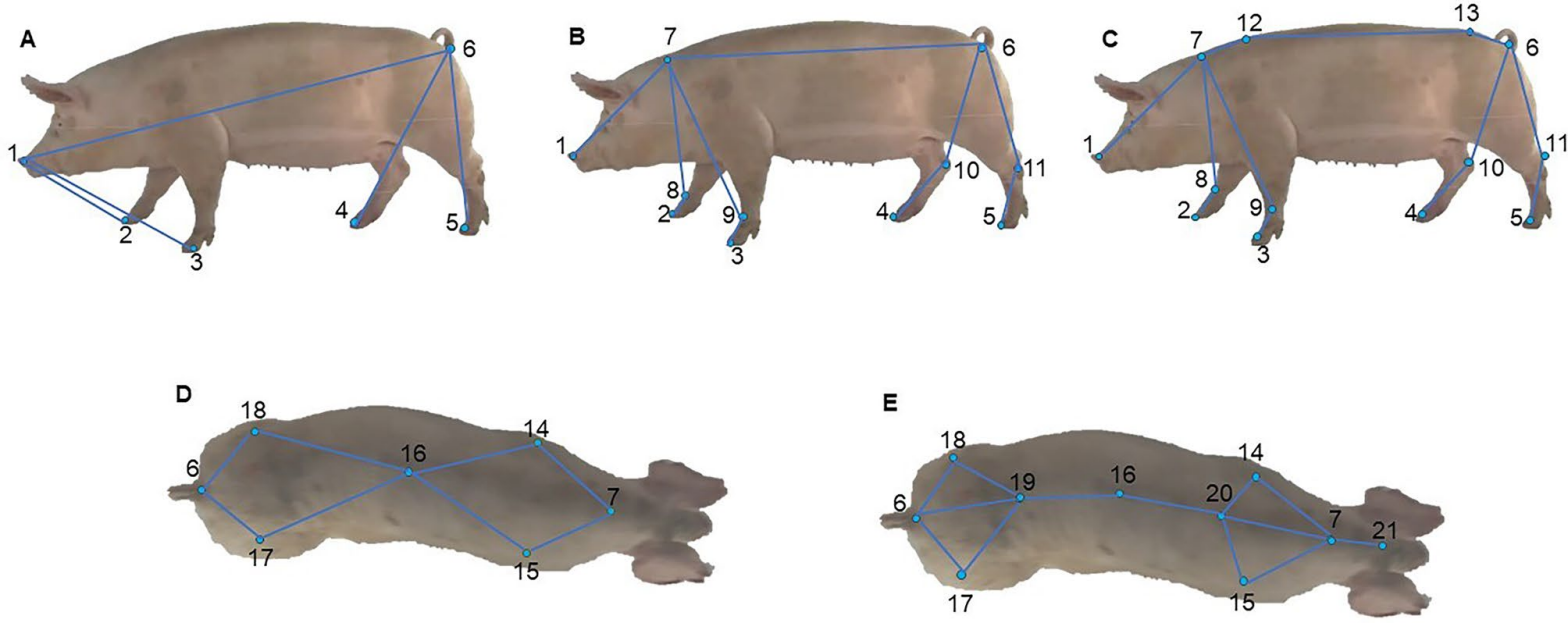
Identification of sows skeletons lateral and dorsal views in SLEAP software. (**A**) (6 keypoints), (**B**) (11 keypoints) and (**C**) (13 keypoints) = Lateral view sow. (**D**) (7 keypoints) and (**E**) (10 keypoints) = Dorsal view sow. Keypoints: (1) snout, (2) hoof front right, (3) hoof front left, (4) hoof posterior right, (5) hoof posterior left, (6) rump, (7) neck, (8) pastern right, (9) pastern left, (10) hock right, (11) hock left, (12) dorsal neck, (13) dorsal rump, (14) scapula left, (15) scapula right, (16) middle, (17) pelvic right, (18) pelvic left, (19) lumbar, (20) thoracic, (21) head.

The SLEAP software was input with a total of 106 2D videos in both the lateral and dorsal views, with 33 videos for each locomotion score of 0, 1, and 2, and seven videos for a locomotion score of 3 due to the low number of animals with this score. Initially, the framework was defined as a skeleton related to a set of keypoints that were marked on the animal's body in each frame of the video. The lateral skeleton was defined by 13 keypoints: snout, neck, right and left hock, right and left metacarpal, dorsal neck, dorsal tail, rump, and hoof right, left, front and posterior (Fig. 4c). To simplify the model, a lateral skeleton with 11 keypoints was also defined, where the dorsal neck and dorsal tail keypoints were removed (Fig. 4b). For the dorsal view, a skeleton with 10 keypoints was created: neck, scapula right/left, spine middle, pelvic right and left, tail, head, thoracic and lumbar (Fig. 4e). A preliminary analysis was performed on the established keypoints to identify variations. Based on this analysis, a lateral skeleton with 6 keypoints, removing the dorsal neck, dorsal tail, right and left hock, neck and right and left metacarpal keypoints (Fig. 4a) and a dorsal skeleton with 7 keypoints, further removing the head, thoracic, and lumbar keypoints (Fig. 4d) were established. There were 20,323 frames in the lateral videos, of which 3293 frames were manually labelled by two trained individuals. There were 14,537 frames in the dorsal videos, of which 2311 frames were manually labelled. Only the frames in which the sows were fully visible in the videos were considered for training and testing the models.

The keypoints were defined to identify and analyse movements for future kinematic studies and to relate these movements to the sow's locomotion score, determined by the panel of observers. The snout and neck keypoints were chosen to identify compensatory head movements. The neck dorsal, tail dorsal, neck, and tail keypoints were chosen to identify spine arching. The hocks, hoots, and metacarpals keypoints were chosen to identify which limb the sow had difficulty walking on^34^.

The SLEAP software allows for the settings to be customized to improve the computational model according to the project’s needs. Nineteen models (Table 4) with 6, 7, 10, 11 and 13 keypoint skeletons were developed using 5 different convolutional neural network (CNN) architectures: LEAP, U-Net, ResNet-50, ResNet-101, and ResNet-152.

- LEAP (Estimates Animal Poses): LEAP's pose estimation architecture is based on deep learning and uses a 15-layer convolutional neural network to predict the positions of animal body parts^40^.
- U-Net: U-Net is a convolutional neural network (CNN) architecture with 23 layers and a "U"-shaped format^41^. The presence of both an encoder and a decoder in this architecture helps it address complex tasks such as posture classification^42,43^.
- ResNet-50: ResNet-50 is a 50-layer residual neural architecture trained on the ImageNet image database; it is an improved version of the CNN^44^.
- ResNet-101 and ResNet-152: ResNet-101 and ResNet-152 are residual neural architectures with 101 layers and 152 layers, respectively^44^.

Figure 4a–e, as well as pixel error graphics, were generated with SLEAP software. The videos of sows with labelled keypoints (ground truth) and unlabelled keypoints (predicted by the algorithm) were developed in the SLEAP software. With these data, a video could be developed with only the *x* and *y* coordinates in pixels and without the animal by using the script created in MATLAB R2021b (Mathworks Inc., USA). The videos are provided in the supplementary material (Supplementary Videos 1 and 2). The general and specific hyperparameters in the 19 models for lateral and dorsal views, such as input scale, epochs, batch size, initial learning rate, Gaussian noise, and rotation, were configured. The split method was used for model evaluation, where the videos images repository was randomly split into 85% for training and validation and 15% for testing.

### Metrics for evaluating computational models

Pose estimation is a difficult activity for the algorithm to perform, as it involves variations in lighting, perspective projection, and the occlusion of portions of images^39,45^. The model’s evaluation is complex because it involves many errors that affect the performance of the algorithm^45,46^. In addition, the average precision (AP) metric cannot interpret the behaviour of the algorithm and does not identify all the existing errors^45,46^. Due to some limitations in pose estimation analysis, new metrics for the task have been developed, such as object keypoint similarity (OKS), mean average precision (mAP), and distance average (dist.avg).

The OKS metric was designed to identify different types of errors in pose estimation algorithms; it can be used for estimating the poses of humans and animals. This metric calculates the average similarity between the person's labelled (ground truth) and the unlabelled (predicted by the algorithm) keypoints^45,47^. OKS calculates the true value and predicts the similarity of human keypoints. OKS is defined by Eq. (2):

2 $$OKS= \frac{{\sum }_{i}{\text{exp}}(-{d}_{i}^{2}/2{s}^{2}{k}_{i}^{2})\delta ({v}_{i}>0)}{{\sum }_{i}\delta ({v}_{i}>0)}$$

where ${d}_{i}$ is the Euclidean distance between the detected keypoint and its corresponding ground truth; ${v}_{i}$ is the visibility flag of the ground truth; s denotes the person scale (in our study, this is the sow); and ${k}_{i}$ is a per keypoint constant that controls falloff^22,39^. When the SLEAP software was developed, OKS was employed as a lower bound on the true accuracy of the model because some reference points on animals can be difficult to precisely locate^39^. The distribution of OKS scores and the mAP metric were obtained to summarize accuracy across the dataset.

The mAP is based on object keypoint similarity (OKS). The mAP is the mean value of multiple APs with different thresholds^47^ and is used to measure the average accuracy of pose estimation across multiple individuals^48^. The mAP metric provides an overall evaluation of all the keypoints, so its evaluation is more accurate, as each keypoint has a different scalar^47^. Calculating mAP, which is used to evaluate the pose estimation accuracy in SLEAP, involves considering predicted instances as either true positives (TP) or false positives (FP) based on OKS^39^. The mAP and the mean average recall (mAR) metrics provide balanced estimates of accuracy, yielding reliable precision^39^. AP is defined by Eq. (3)^47^:

3 $$AP= \frac{\sum_{n}\delta \left({OKS}_{n}<T\right)}{{\sum }_{n}1}$$

The distance average (Dist. avg) measures the Euclidean distance between the ground truth and model predictions based on the algorithm and architecture of each skeleton keypoint^45,49^. Thus, in the labelling and training phases of the neural network, ground truth labels were provided to identify body part positions in the images^40^.

A variation on the use of Euclidean distance as a metric is the percentage correct of keypoints (PCK), which assesses the accuracy of joint localization^47^, has also been used; PCK is used to indicate whether each keypoint prediction is correct^47^. The PCK metric has been used in other studies as well in human^50,51^ and animal^47^ pose estimation tasks. The PCK is calculated using Eq. (4):

4 $${PCK}_{i}=\frac{{\sum }_{n}\delta (\frac{{d}_{{n}_{i}}}{{s}_{n}}<\alpha )}{{\sum }_{n}1}$$

where $n$ is the $n$ th target; ${d}_{{n}_{i}}$ is the Euclidean distance between the $i$ th predicted keypoint and its ground truth of the $n$ th target; $\alpha$ is a constant parameter that controls the relative correctness of the evaluation; and ${s}_{n}$ is a normalized scalar of the $n$ th target. $\alpha$ and $s$ vary among different works.

In addition, pixel errors were analysed for each of the labelled reference points on the animal's body.

## Results

### Data repository

A repository was created with 281 lateral videos images and 237 dorsal videos images separated by sow locomotion scores with 75% and 100% evaluation confidence. Technical problems in the acquisition of the images compromised de number of dorsal images, as the selection criteria was based on the agreement among evaluators, who only assessed lateral views. Given the fact that, 20.67% lateral videos images and 29.46% dorsal videos images had less than 75% of the assessment confidence, by the evaluators, they were excluded from the tested dataset. Each video was approximately 30 s long and in MP4 format (Table 3).

**Table 3:** Evaluation confidence of videos with lateral and dorsal views in different locomotion scores.

Views	Scores	Assessment confidence		
		75%	100%	
Lateral	0	28	131	Number of the videos
	1	30	43	
	2	19	23	
	3	4	3	
Dorsal	0	27	97	
	1	26	40	
	2	19	21	
	3	3	4	

In this work, only one dataset was created with lateral and dorsal videos. The dataset was used to evaluate the locomotion scores by the experts and part of it was used to develop the models.

Finally, a total of 106 pairs of videos, from the existing repository, evaluated by assessors, were selected as a data set for the development of the models.

The video repository with the videos images separated by locomotion score is open access and is available on the Animal Welfare Science Hub website https://www.animalwelfare-hub.com/video-repository.

The videos with the labeled keypoints are not available.

### Modelling

Simulations of hyperparameter changes were carried out in order to demonstrate how the data set can be used for animal analysis, as described in Table 4. Similarly, specific hyperparameters were selected, as described in Table 4. According to the data presented in Table 5, obtaining high accuracy (mAP) and similarity (OKS) values between the ground truth and predicted values, as well as a low distance between the labelled and predicted keypoint (dist.avg), was possible. In addition, the accuracy of the correct keypoints is within the distance limit between predicted and labelled keypoints. Skeleton models with 10, 11 and 13 keypoints were tested. The best skeletons were subsequently created with 6 and 7 keypoints, these being the lateral 1 and dorsal 5 models.

**Table 4:** Configuration of general and specific hyperparameters in each model of the lateral and dorsal views for the different keypoints from the pig skeleton.

Model	View	Skeleton (number of keypoints)	Architecture	Hyperparameters			
				Specific		General	
1	Lateral	6					
2		11		Max stride	8		
3		13	LEAP	Filters	64	Input scaling	0.25
4	Dorsal	7		Filters rate	2	Epochs	200
5		10				Initial learning rate	0.0001
6	Lateral	6	U-Net	Stem stride	none	Gaussian noise mean	0
7		11		Max stride	16	Gaussian noise standard deviation	5
8		13		Filters	16	Rotation maximum angle	5
9	Dorsal	7		Filters rate	2	Rotation minimum angle	-5
10		10		Middle block	Activated		
				Interpolate activated	Activated		
11	Lateral	11		Weights frozen	Activated		
12		13	Res-Net50	Method interpolation	Activated		
13	Dorsal	10		Block stride	2		
14	Lateral	11		Filters	64	Input scaling	0.25
15		13	Res-Net101	Filters rate	1	Epochs	200
16	Dorsal	10		Refine convolutional layer	2	Initial learning rate	0.0001
17	Lateral	11		Batch norm activated	activated		
18		13	Res-Net152	Transposed Conv kernel size	4		
19	Dorsal	10		Max stride	32		

Batch size: number of samples processed before the model is updated; Epoch: the number of complete passes through the training dataset; Initial Learning Rate: how quickly the model is adapted to the problem; Rotation Angle (min/max): image rotation angle; Gaussian noise adds randomness to the input data or weights.

**Table 5:** Comparison of precision metrics in each model of the lateral and dorsal views generated using the different keypoint skeletons.

Model	Skeleton (number of keypoints)	Views	Architectures	Precision metrics			
				OKS	mAP	Dist.avg	PCK
1	6	Lateral		0.94	0.90	6.83	0.51
2	11			0.93	0.89	7.86	0.44
3	13		LEAP	0.93	0.88	8.25	0.43
4	7	Dorsal		0.84	0.69	12.53	0.25
5	10			0.86	0.72	11.37	0.28
6	6	Lateral		0.93	0.89	7.45	0.48
7	11			0.92	0.88	8.07	0.45
8	13		U-Net	0.92	0.87	8.71	0.41
9	7	Dorsal		0.78	0.57	13.28	0.28
10	10			0.80	0.63	11.62	0.26
11	11	Lateral		0.41	0.01	34.99	0.05
12	13		ResNet-50	0.76	0.51	19.03	0.14
13	10	Dorsal		0.39	0.01	26.17	0.03
14	11	Lateral		0.52	0.10	28.80	0.08
15	13		ResNet-101	0.54	0.11	26.79	0.08
16	10	Dorsal		0.50	0.11	26.03	0.05
17	11			0.61	0.23	23.00	0.10
18	13	Lateral	ResNet-152	0.51	0.06	31.52	0.09
19	10	Dorsal		0.50	0.10	22.90	0.07

*OKS* Object keypoint similarity, *mAP* mean average precision, *Dist.avg* distance average; Correct Percentage of Keypoints (PCK).

The results in Table 5 indicate that the models with the ResNet-50, ResNet-101, and ResNet-152 architectures were inferior to the models with the LEAP and U-Net architectures. Therefore, the models with 6 and 7 keypoints skeletons were not trained with the ResNet architectures on the lateral and dorsal views.

**Figure 5:**
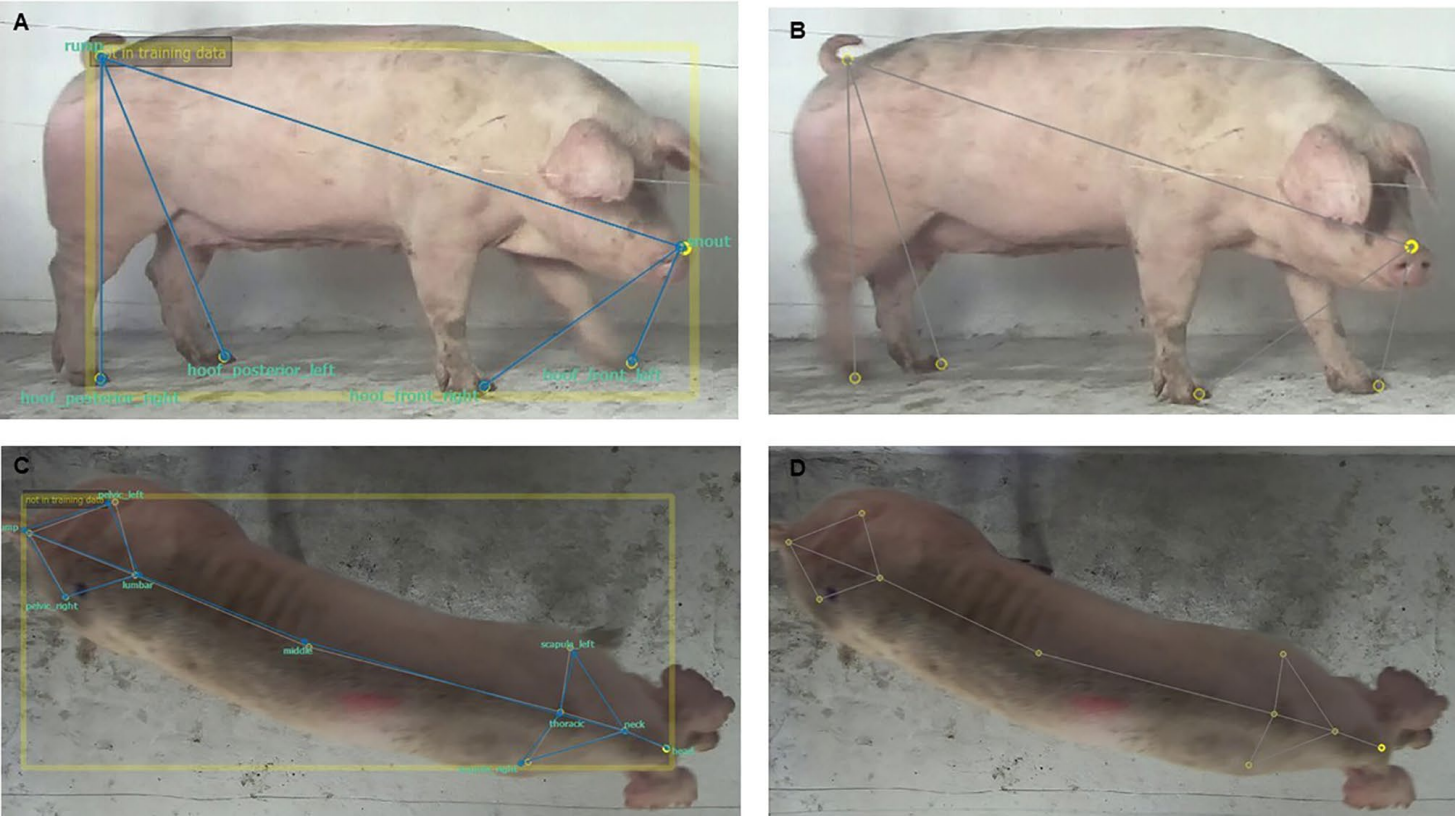
Lateral and dorsal videos screenshots of the keypoint detection results. (**A**) visão lateral com os keypoints nas cores amarelo (predito pelo software) e azul (ground truth). (**B**) visão lateral com os keypoints na cor amarelo (predito pelo software). (**C**) visão dorsal com os keypoints nas cores amarelo (predito pelo software) e azul (ground truth). (**D**) visão lateral com os keypoints na cor amarelo (predito pelo software).

Figure 5 is a representation of the posture detection sequence in lateral and dorsal videos after the training stage. For a better view of the video, see the supplementary material.

**Figure 6:**
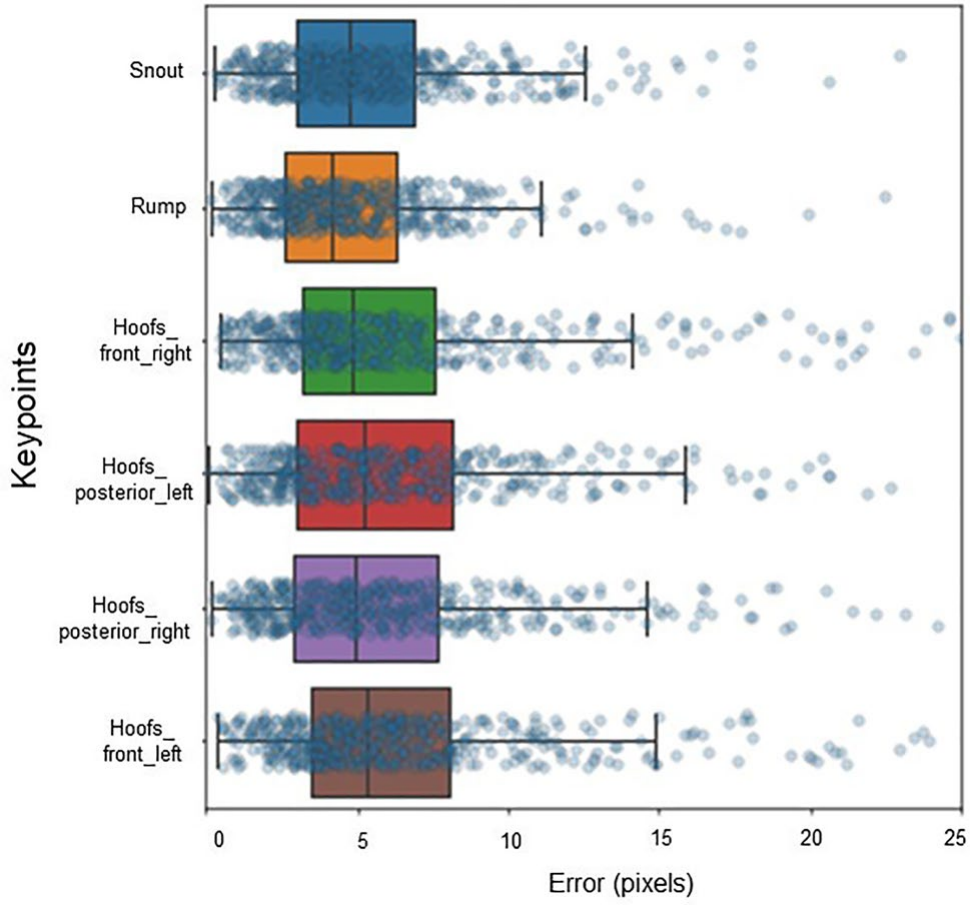
Error in pixels of each keypoint with the 06-keypoint sow skeleton of model 1 using the LEAP architecture and the lateral view.

**Figure 7:**
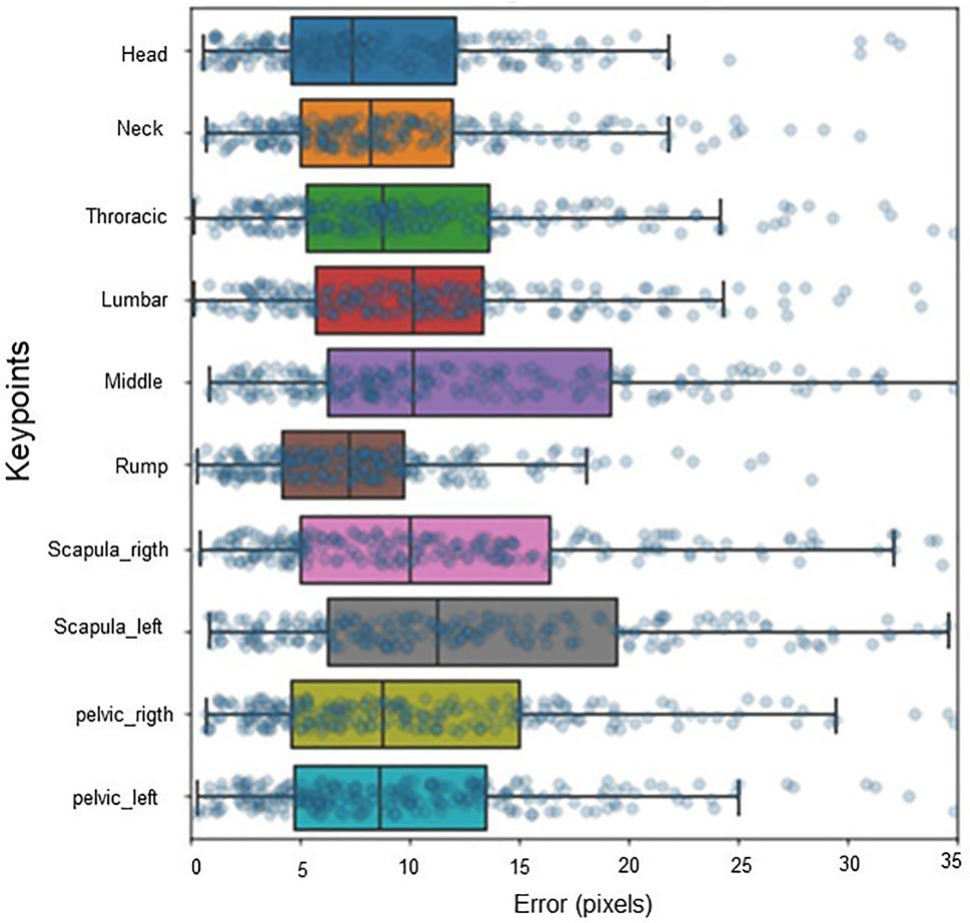
Error in pixels of each keypoint with the 10-keypoint sow skeleton of model 5 using the LEAP architecture and dorsal view.

Precision was analysed by comparing each model using the OKS, mAP, and dist.avg metrics, the PCK (Table 5), and the errors per pixel for each keypoint labelled on the sows (Figs. 6 and 7). Figures 6 and 7 show the differences in keypoint identification based on the pixel error dispersion marked in all the videos trained with the LEAP and using the two best models.

Based on the presented results, we have successfully achieved the goal of building computational models to identify sequences of lateral and dorsal poses in videos with different locomotion scores of sows. Currently,he project is using these results to obtain and to study the kinematic report of pose detections over time, in order to relate it to the locomotion score assessed by the experts.

## Discussion

Models 1 and 5 (Table 5), which both utilized the LEAP architecture with different skeletons, performed better than the other 17 models. This was indicated by their high OKS and mAP values, which are close to one, indicating high pose estimation accuracy^48^. Furthermore, (Dist.avg) between the ground truth and the predictions was low, indicating that the predictions were close to the ground truth. Additionally, hyperparameters such as the degree of image rotation and Gaussian noise (Table 4) were adjusted to artificially increase the number of images in training and make the model more robust^52^. However, the models using the ResNet architecture and different skeletons achieved results below those of the other models, as evidenced by the low OKS and mAP values and the high Dist.avg value between the ground truth and predictions.

Studies that have used ResNet-50 and ResNet-101 indicated that both architectures are more accurate because they had deeper layers and achieved better results than shallower layers^53^, providing advanced feature representations for a wide range of images^44^. These architectures have been used in tasks such as monitoring sow lactation and piglet movement^54^ as well as in classifying microscopy images^55^. However, in our study, the Res-Net architecture did not show superior results to the LEAP and U-Net architectures, which might be due to overfitting or saturation of precision leading to degradation^53^. We argue that the complexity of the architecture, coupled with the variability in training parameters, can contribute to the occurrence of overfitting, driven by the memorization capacity of the training data. The likelihood of saturation increases with the depth of layers within the architecture. In light of this, it is worth noting that the ResNet 50, ResNet 101, and ResNet 152 architectures possess greater depth and complexity when compared to the LEAP and U-Net architectures.

The LEAP architecture outperformed the other architectures, probably because it was designed to use probability distribution to locate each part of the animal's body in the image and predict the animal’s poses^40^. Methods for analysing animal behaviour or movement usually require many labelled keypoints; however, deep learning-based analysis methods are flexible with respect to the number of labelled keypoints^56^. The locomotor behaviour of sows on skeletons labelled with 6, 10, 11 and 13 keypoints was analysed.

The PCK metric has a distribution threshold between 0.05 and 0.3, which is 0.05 highly accurate for locating the keypoint^47^. Therefore, models that have PCK values within this threshold indicate that they will have high accuracy in identifying the keypoint on the animal's skeleton. For example, model 1 had the highest PCK value compared to all the models. As such, it demonstrated high accuracy in correctly locating the predicted keypoints in relation to the labelled (ground truth) keypoints on the animal's skeleton. As shown in Fig. 6, the anterior and posterior left Hoofs keypoints exhibited greater degrees of error and variation in the pixel results than the anterior and posterior right hoots keypoints. During the sows' locomotion, the keypoints on the left hoof were sometimes occluded. Something similar occurred in the study of Wang et al*.*^34^, where the keypoint of the hoof was very important for behavioural analysis and pig movement identification.

Figure 7 shows that the middle and scapula left keypoints exhibited the greatest degree of error and variation in the pixel results. Artificial intelligence has difficulty comprehending the middle keypoint because it has no specific features when compared to the rump keypoint, which has a specific feature. The scapula left keypoint may have had difficulty distinguishing between the colour of the sow and the colour of the wall due to the similarity between these colours. The same issue occurred with some of the keypoints in the study conducted by Wang et al.^34^.

Data collection in this study was simpler and faster than in other studies. This can be considered an advantage over the SowSIS system^28,29^, which requires installing plates and a ramp, a long period of data collection, adapting the animals, and installing equipment. However, keeping the data collection location clean was challenging for the authors of^28,29^ and for this work. All these studies, including the present one, required locomotion experts to evaluate the videos and a manual labelling team to train the model. Creating the repository of labelled real images was essential for training the computer vision model^57^. The lack of good quality image repositories for training and testing models in the algorithm development phase is a limitation^58^. Moreover, manually labelling all the videos is time-consuming and laborious^56,57^. Thus, our results indicate that the data repository that was made available through this research, could be used as a research and teaching tool for professionals, teachers and students to evaluate sow locomotion in a practical way. Based on the results, using the LEAP architecture with skeletons of six keypoints in the lateral view and 10 keypoints in the dorsal view for pose estimation in sow locomotion using deep learning showed favourable results. These architectures achieved accuracies of 0.90 and 0.72, average distances of 6.83 and 11.37, and similarities of 0.94 and 0.86, for the lateral and dorsal views respectively. Our computer model was able to identify the animal’s pose accurately and precisely.

The results presented in this manuscript indicated that the study has offered data to promote further kinematic studies to help in the early detection of lameness in sows, automatically. A novel contribution is the fact that we demonstrate that, it was possible to obtain a lateral model and a dorsal model, from the same sow, at the same time. The development of lateral and dorsal models was studied, because in pig farms the use of an imaging system at height of the animals is very challenging. Curious animals can damage the equipment and injure themselves. Also, if the system is used in a group housing, one animal can be occluded by another. For these reasons, the dorsal model is promising.

The proposed PLF tool can be useful on farms where some management is done individually. The computational system could be used to evaluate sows when they are subjected to management practices. Animals could be evaluated, individually, when they are moved to the farrowing house, out of the farrowing house, for example. On the farm where the video image database was obtained, the sows are handled individually from the stalls, after mating, to the pen. The study presented demonstrates that it is possible to use a deep learning tool to identify keypoints on the animal's body, which are related to lameness detection. In addition, by demonstrating promising results, there is the possibility of expanding to establish lameness detection models with several animals, in several scenarios.

The biggest challenges in this study were to obtain a balanced database of video images including a good representation of all locomotion scores, especially scores 2 and 3. In addition, labelling the lateral and dorsal videos was laborious and time-consuming. Training the models was very time-consuming, especially the ResNet 50, ResNet 101 and ResNet 152 models, which took around 20 h. The LEAP and U-Net models took around 8 h.

## Conclusion

Computational models have the potential to automatically identify and to estimate locomotion poses in sows. The results showed high values of similarity (OKS), average precision (mAP) and accuracy of the location of keypoints (PCK) and low distance between the ground truth keypoints and the predictions (average distance). The purpose of these models is to be used as a tool and a method for precision livestock farming that can help objectively identify the locomotion behaviour of sows, identifying lameness at early stages, to promote rapid interventions, improving animal welfare. In the repository of 2D video images with different locomotion scores contributed to the development of this work and is available to support other research and teaching activities related to sow locomotion.

### Supplementary Information

Supplementary Video 1.

Supplementary Video 2.

Supplementary Information.

## Data Availability

The data supporting the findings of this paper are available from the corresponding author upon reasonable request.
