# Supplementary material for: Deep learning pose detection model for sow locomotion
Source: Sci Rep. 2024 Jul 16;14:16401. doi: 10.1038/s41598-024-62151-7 (PMC11252330; doi:10.1038/s41598-024-62151-7)
Supplement: Supplementary file 3 — Supplementary Information. [file 41598_2024_62151_MOESM3_ESM.docx]

Labelling of supplementary material 1 and 2

Supplementary material 1: demonstration of the identification of keypoints in the side-view video of an animal with a locomotion score of 2 in the SLEAP software.

The yellow keypoints represent the predicted keypoints and the blue keypoints indicate the manually labeled keypoints.

Supplementary material 2: demonstration of the identification of keypoints in the dorsal view video of an animal with a locomotion score of 2 in the SLEAP software.

The yellow keypoints represent the predicted keypoints and the blue keypoints indicate the manually labeled keypoints.
